# Supplementary figures and images for: Fluctuating reproductive isolation and stable ancestry structure in a fine-scaled mosaic of hybridizing Mimulus monkeyflowers
Source: PLoS Genet. 2025 Mar 31;21(3):e1011624. doi: 10.1371/journal.pgen.1011624 (PMC11978108; doi:10.1371/journal.pgen.1011624)

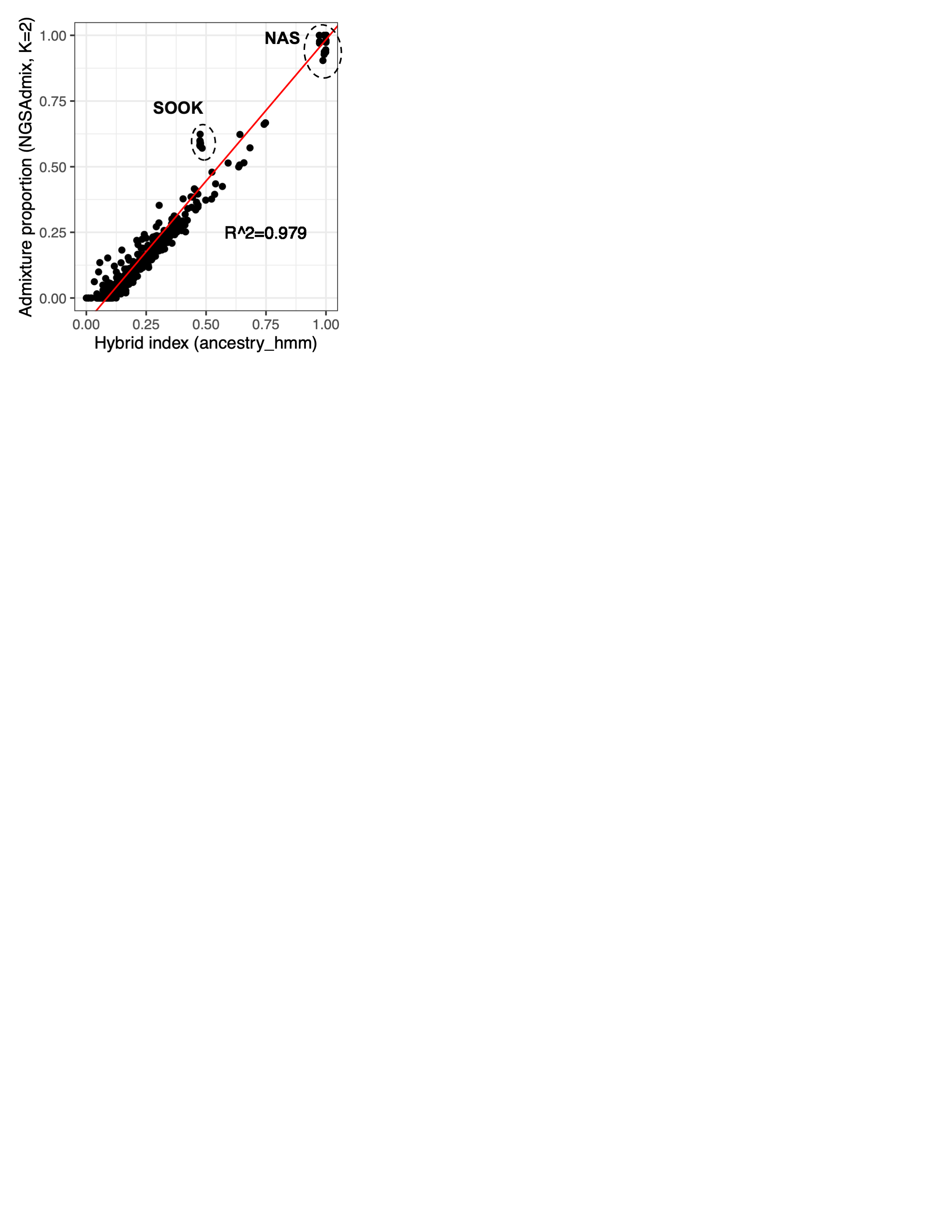

Supplement: S1 Fig — NGSAdmix value is the proportion assignment to one of two clusters by NGSAdmix with K=2; hybrid index is the proportion of sites with M. nasutus ancestry from local ancestry inference using Ancestry_HMM. The circled ‘SOOK’ cluster that deviates from the 1–1 line is composed of M. sookensis polyploid individuals (see Materials and Methods). ‘NAS’ = M. nasutus. (TIFF) [file pgen.1011624.s001.tiff]

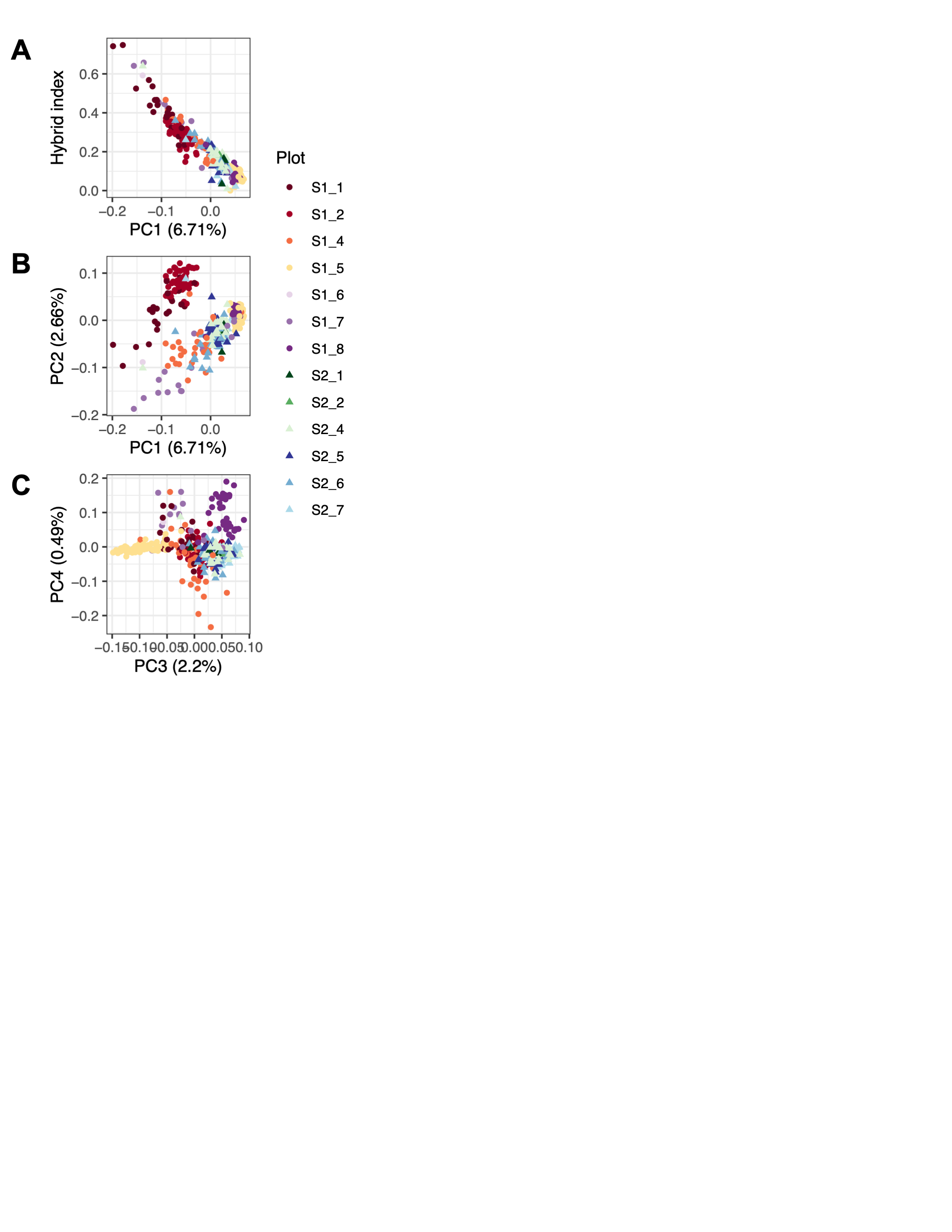

Supplement: S2 Fig — Ancestry_HMM hybrid index and ancestry heterozygosity outputs for A) maternal and B) offspring individuals. Hybrid index or HI is the proportion of ancestry-informative sites called with M. nasutus ancestry from Ancestry_HMM. Ancestry heterozygosity or AH is the proportion of ancestry-informative sites called has heterozygous (out of the total number of called ancestry-informative sites). First-generation hybrids between 100% M. guttatus and 100% M. nasutus would have an expected HI=0.5 and AH=1.0; their offspring would be expected to have lower ancestry heterozygosity (~0.5 if selfed). The circled ‘SOOK’ cluster highlights a group of polyploid M. sookensis maternal plants; their offspring have similarly high AH values, which indicates fixed heterozygosity (polyploidy) rather than diploid F1 status. (TIFF) [file pgen.1011624.s002.tiff]

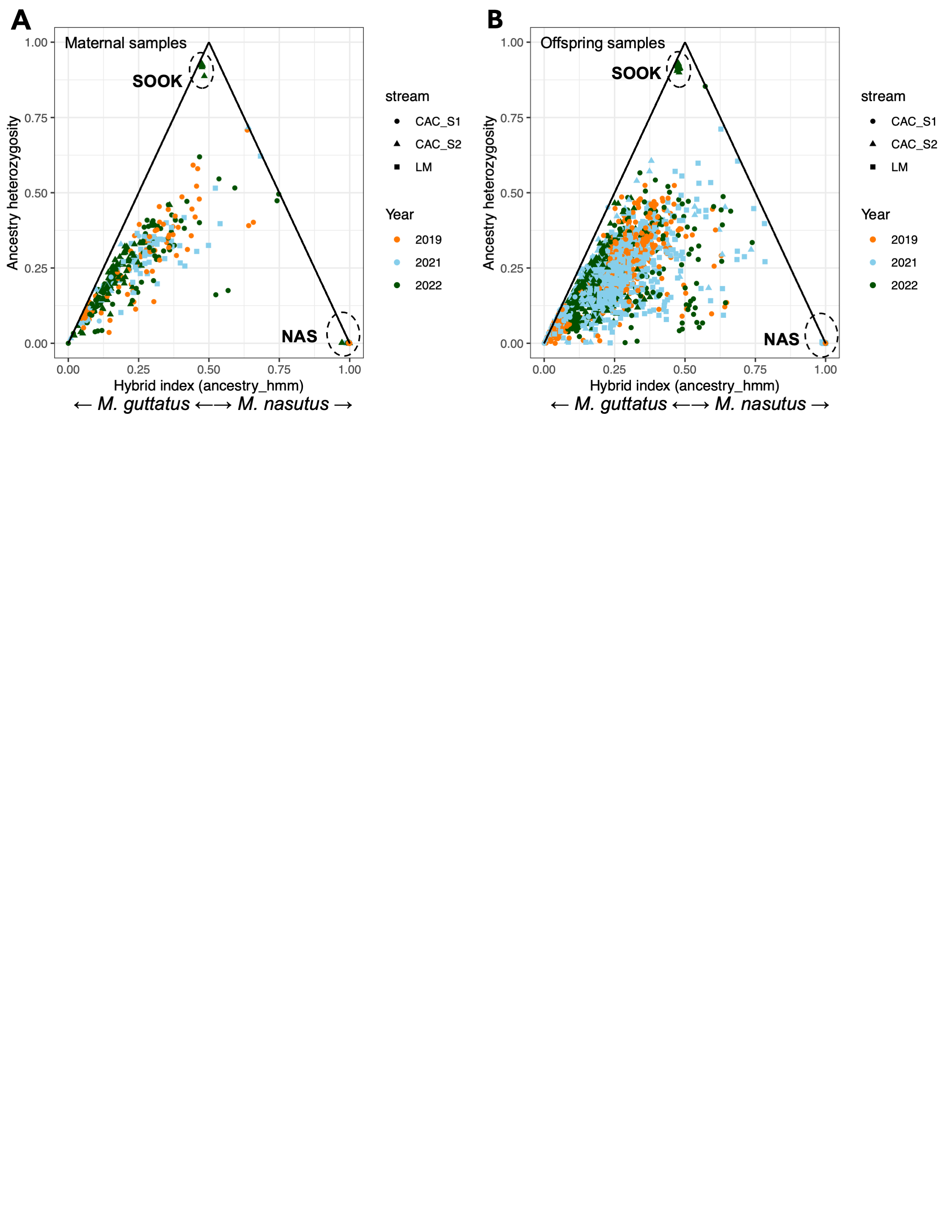

Supplement: S3 Fig — Principal components 1 and 5 from PCAngsd analysis (from the same analysis as PCs 1–4 shown in Fig 2A and 2B). PC1 correlates strongly with M. guttatus vs. M. nasutus ancestry (Fig 2B). PC5 clearly delineates a group of individuals identified as the polyploid species M. sookensis. (TIFF) [file pgen.1011624.s003.tiff]

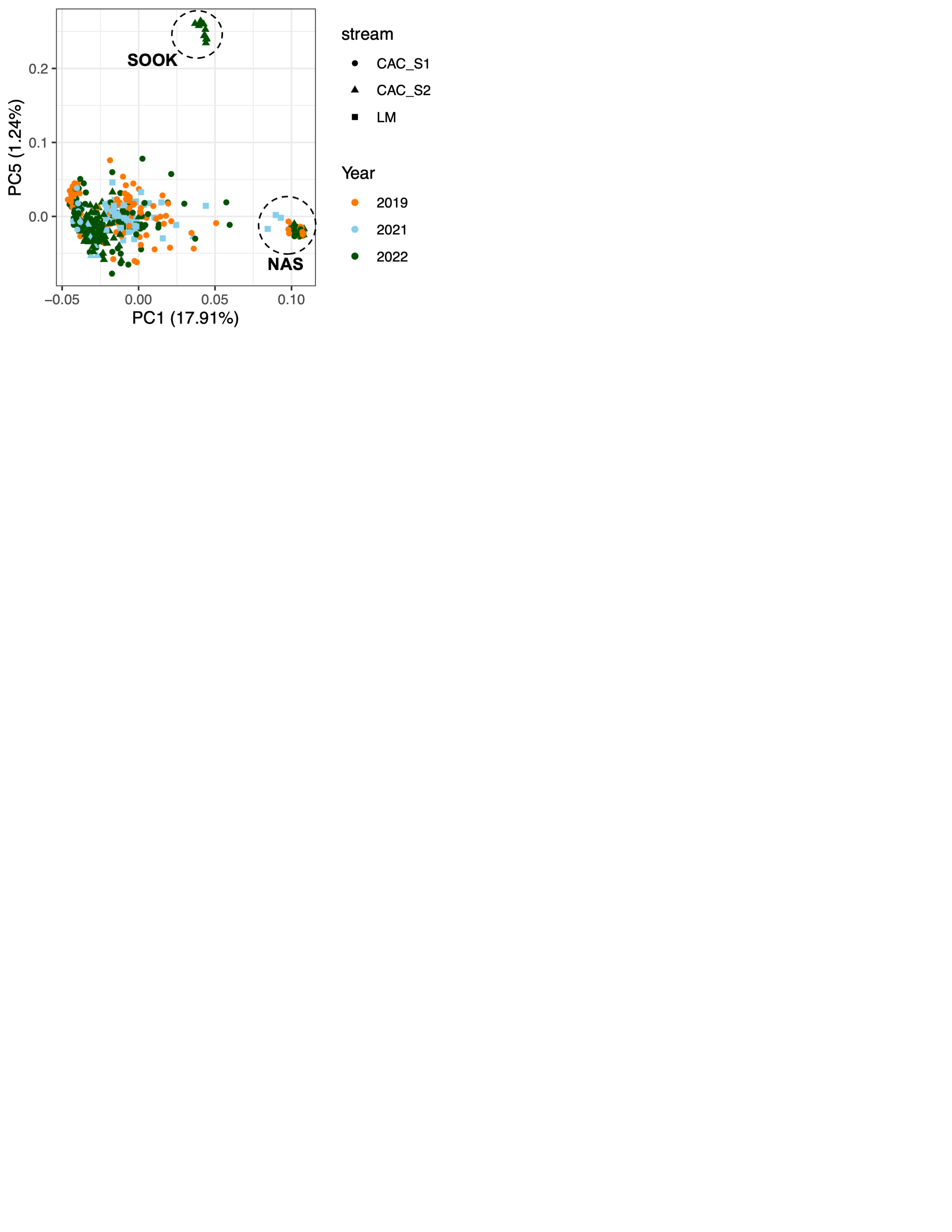

Supplement: S4 Fig — A) PC axis 1 vs. hybrid index, demonstrating that ancestry still drives the major axis of genomic variation in the dataset even without M. nasutus samples. B) PC axes 1 and 2, demonstrating some separation of certain CAC_Stream1 plots (particularly S1_1 and S1_2) from the rest of the data. C) PC axes 3 and 4, showing further variation within CAC_Stream1 that is partitioned by plot identity. Note that CAC_Stream2 is not differentiated from CAC_Stream1 in these first four axes, but the major axes of variation instead separate variation within CAC_Stream1. LM samples, M. nasutus samples, and M. sookensis samples were excluded prior to running thi PCA. (TIFF) [file pgen.1011624.s004.tiff]

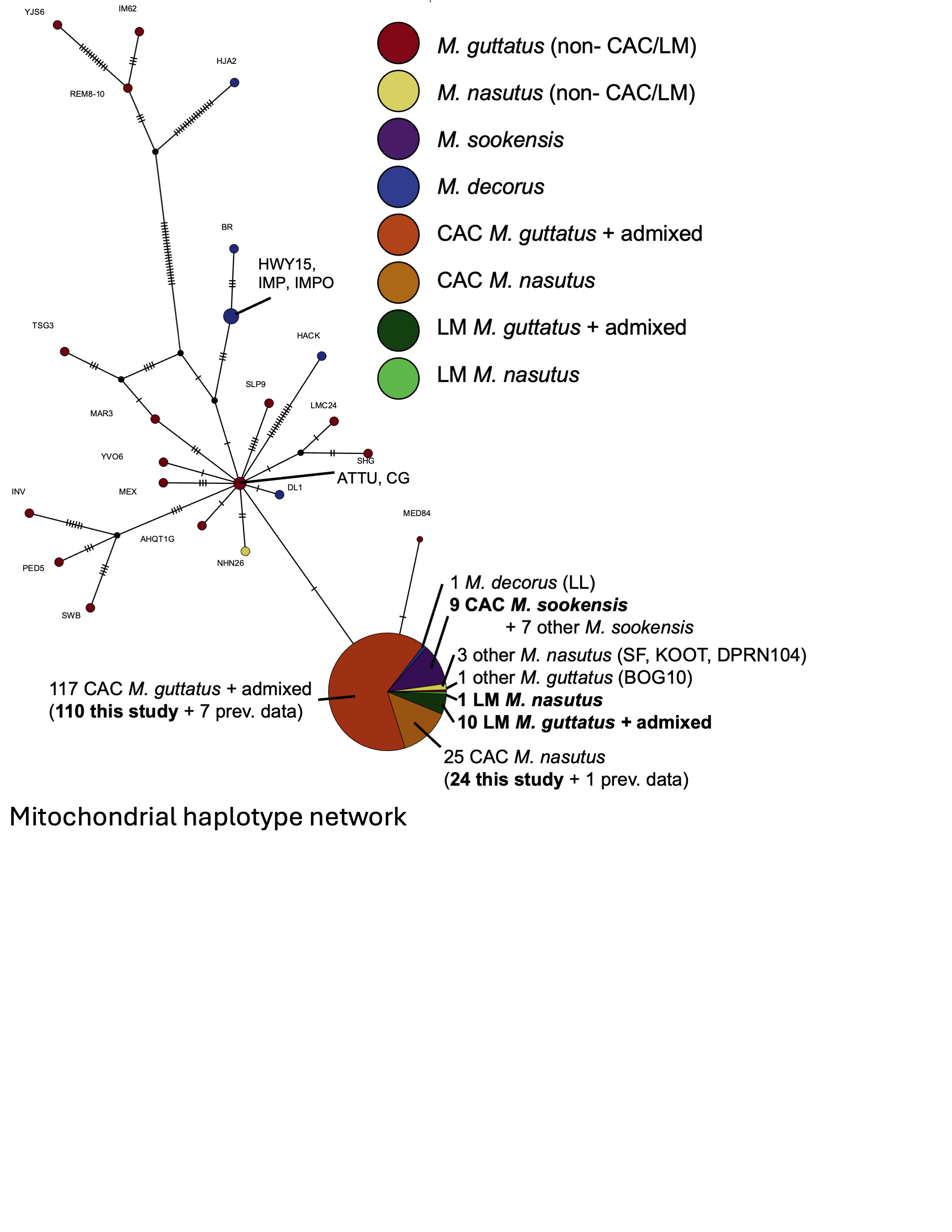

Supplement: S5 Fig — . NJ-Net haplotype network of mitochondrial variation built from 154 CAC and LM maternal samples sequenced in this study (bolded captions) and 49 previously sequenced samples (non-bolded captions) from the M. guttatus species complex [98], using 120 total variant sites (39 parsimony-informative). The mitochondrial network largely agrees with the chloroplast network (Fig 3), with a single haplotype present in all maternal samples from Catherine Creek and Little Maui, including M. guttatus, admixed, M. nasutus, and M. sookensis samples. All other M. nasutus samples from across the range share this haplotype or a close derivative, as do samples from M. sookensis (a polyploid with M. nasutus as maternal parent). M. guttatus haplotypes are more variable, with only one sample not from CAC or LM sharing the M. nasutus haplotype. M. decorus is another member of the M. guttatus species complex with variable mitochondrial haplotypes. (TIFF) [file pgen.1011624.s005.tiff]

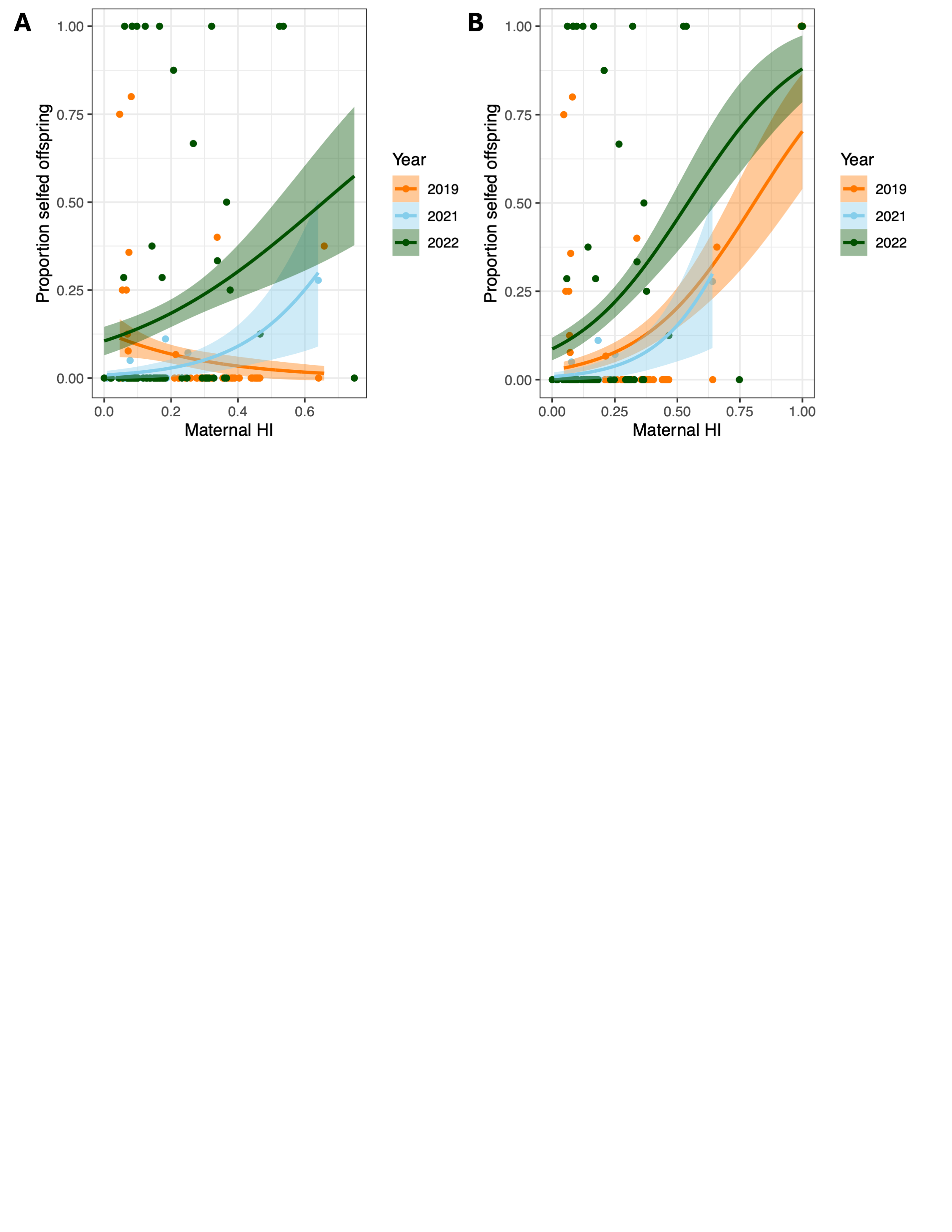

Supplement: S6 Fig — Proportion of selfed vs. outcrossed offspring per maternal family vs. maternal hybrid index (proportion M. nasutus ancestry of the maternal plant), showing (A) a binomial model fit including M. nasutus maternal families, and (B) a binomial model fit excluding M. nasutus families. Self vs. outcross determined by the BORICE Bayesian model. Offspring without>=90% posterior probability of either state were removed. Lines and shaded regions indicate model fits and 95% confidence intervals from a binomial GAM regression using stream and year as additional factor variables (S2 Table). (TIFF) [file pgen.1011624.s006.tiff]

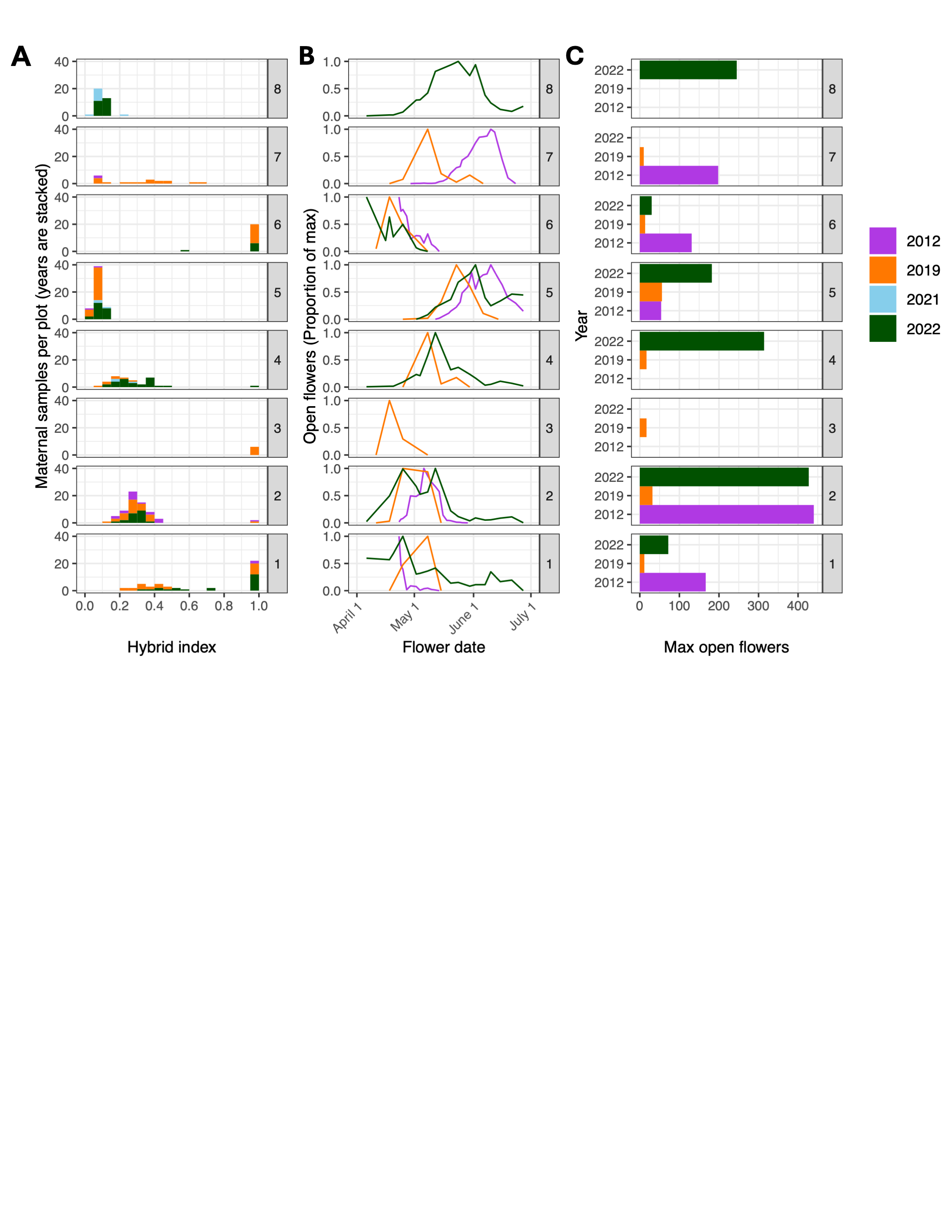

Supplement: S7 Fig — A) Histograms of binned hybrid index for sampled maternal plants in each CAC_Stream1 plot, with years as stacked colors. 0=M. guttatus, 1=M. nasutus. B) Phenology (counted open flowers) throughout the flowering season in seven plots within CAC_Stream1 during the 2012, 2019, and 2022 seasons, scaled to the maximum number of open flowers counted within that plot in that year. Plots with primarily M. nasutus individuals (plots S1_1,S1_3, and S1_6) had early flowers, plots with more admixed individuals (plots S1_2, S1_4, and S1_7) had intermediate peak flowering, and plots with primarily M. guttatus (plots S1_5 and S1_8) had later peak flowering. Peak flowering was similar but not identical across years. Tails of open flowers indicate that in 2022, plots with admixed individuals (plots S1_1,S1_2, and S1_4) continued flowering for much longer than in 2019, resulting in more overlap with M. guttatus plots (plots S1_5 and S1_8). C) Maximum number of open flowers on any one day for each plot. In 2019, there were far fewer open flowers throughout the growing season than in 2021 or 2022. (TIFF) [file pgen.1011624.s007.tiff]

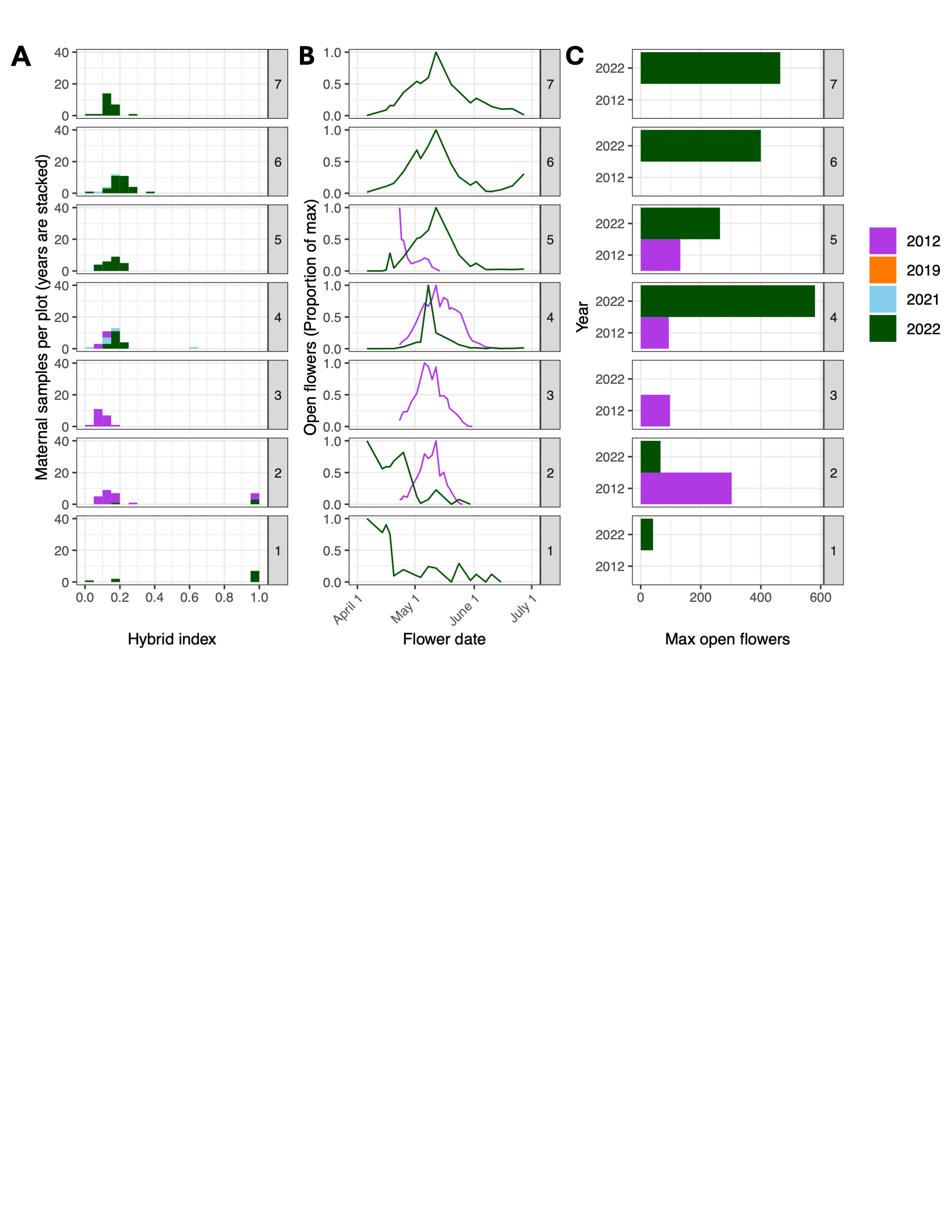

Supplement: S8 Fig — D) Histograms of binned hybrid index for CAC_Stream2 plots. E) Phenology (counted open flowers) for CAC_Stream2 plots, scaled to the maximum number of open flowers counted within that plot in that year. F) Maximum number of open flowers for CAC_Stream2 plots. (TIFF) [file pgen.1011624.s008.tiff]
