# Supplementary material for: Fluctuating reproductive isolation and stable ancestry structure in a fine-scaled mosaic of hybridizing Mimulus monkeyflowers
Source: PLoS Genet. 2025 Mar 31;21(3):e1011624. doi: 10.1371/journal.pgen.1011624 (PMC11978108; doi:10.1371/journal.pgen.1011624)
Supplement: Attachment — Submitted filename: Response_to_reviewers_PLOS_Genetics_DEC2024.docx [file pgen.1011624.s015.docx]

**Dear Dr. Lasky,**

Thank you for the helpful comments on our manuscript. We appreciate the detailed and thoughtful comments of both reviewers, which we have incorporated into a revised version of the manuscript. In response to the reviewers’ suggestions, we have made some minor changes to methodology and analyses, which are also reflected in associated changes to the results. In addition, we have clarified our language throughout the manuscript to describe our sampling scheme more transparently, emphasize the concrete results we have obtained, and distinguish between the direct conclusions of our data and more speculative hypotheses for future study. We believe the revised manuscript is now easier to follow, more complete in its treatment of the data, and more straightforward about the conclusions we can draw from this study.

One major change we made, suggested in particular by Reviewer 2, is to focus on more completely analyzing each individual stream and presenting the analyses of the streams separately where appropriate. This allows us to draw comparisons and contrasts across the streams and make full use of our breadth of sampling, while also clarifying when some results apply to a single stream and not necessarily to all streams. In addition to the text, a few figures and tables have been revised to reflect these changes.

Another substantial change is to de-emphasize certain conclusions of our work regarding the connection between specific environmental factors and variation in reproductive isolation, as well as the link between spatial structure and potential microhabitat variation. As pointed out by the reviewers, these are more speculative in nature and would require follow-up work with additional years or other types of data to fully answer. We still discuss these potential conclusions but reframe them in terms of hypotheses for future research.

In addition to these changes, many minor edits and improvements have been made in response to reviewer suggestions, especially with regard to clarifying our sampling schemes and laying out the justification for methodological choices. Specific responses to each of the reviewers’ suggestions are provided below, with the reviewer comments in italics and our response in blue underneath. We provide line numbers pointing to where changes were made – note that these line numbers refer to the revised manuscript document with tracked changes (the clean revised manuscript has different line numbering). We appreciate your consideration of this revised and improved manuscript for publication in PLOS Genetics.

**Sincerely,**

**Matthew Farnitano, Keith Karoly, and Andrea Sweigart**

***Additional Editor Comments (if provided):***

*I believe the reviewers have made many helpful suggestions. In particular I think it is important to address concerns over sub-setting the samples to particular ('representative') years and locations: how those decisions were made, why subset at all. I also agree that conclusions about microhabitat variation based on the data here do not seem supported.*

Thank you for this thoughtful summary of the reviewer comments. We made a number of changes to address these points, which are detailed in the responses below. We highlight a few key changes here:

Choices about when to focus on one stream vs. all three streams were mostly related to the available data that was collected: multi-year sampling was primarily focused on a single stream, CAC_S1, with a single year of data from LM and only some years sampled in CAC_S2. Phenology data was also not available for 2021 (due to COVID-related constraints on sampling). In the Introduction (lines 157-170) and results (Table 1 and lines 182-187), we added language to clarify these sampling constraints. We highlighted where spatial breadth of sampling was used (all three streams) vs. where depth of sampling (multiple years for a single stream) was used, acknowledging that they provide complementary information. In addition, at the request of the reviewers, we added more analysis and discussion of CAC_S2 and the differences in structure between the streams. For example, Tables 2 and 3 now include analyses split by stream for calculations of selfing rate and reproductive isolation, model fits in Figures 4E and 5A-B are separated by stream, and all statistical models (detailed in Table S2) test for an effect of stream. For phenological isolation specifically, there was concern over choosing a single representative plot for each cohort – we re-analyzed this data including multiple plots per cohort, only excluding two plots that could not be clearly classified into any of the three cohorts (Materials and Methods lines 1320-1328).

Regarding microhabitat conclusions, we modified the language in the Introduction (lines 169-177) and Discussion (834-835, 877, 943-946) to focus on our more direct findings (spatial structure in ancestry and partial isolation between cohorts in CAC_S1). We believe that microhabitat variation is likely important as well, but we clarified in the discussion that this is speculative based on previous work and our own inference, and is not directly addressed by the current study. We highlight this as an avenue for future work in the system (917-935).

We believe these changes to the analysis, along with the other changes detailed in comments below, will address the reviewers’ concerns.

***Reviewers' comments:***

***Reviewer #1:***

*1. Fig.2 shows that CAC_A and CAC_B only contain samples from CAC_S1 stream (open circles) and CSC_C contains both. However, it seems CAC_C tends to have more samples of year 2021 and 2022 while the others tend to have samples of year 2019? And it is not easy to understand “genetic differentiation between samples separated by as few as 50 m” (Line 170) as CAC_S1 and CAC_S2 still shared the same cluster CAC_C in Fig.2 So it would be good to label the three distinct clusters of admixed individuals (microspatial structures) in the map of Fig. 1C. It is quite surprising to observe genetic differentiation within 50 m for the outcrosser M. guttatus. Is there any possibility of severe inbreeding of M. guttatus within a short sampling distance? Please highlight two species in Fig.2 A lower panel.*

Thank you for your comments! You have made us realize that the sampling design was not laid out clearly enough upfront in the results, which led to confusion about the interpretation of Figure 2. We have now provided these details at the start of the Results (lines 183-187) as well as directly in the Figure 2A legend. This hopefully clarifies that PCA cluster ‘CAC_C’ has relatively fewer 2019 samples because it contains stream CAC_S2 samples, and stream CAC_S2 was not sampled in 2019. For similar reasons, Reviewer 2 asked that we move previous table S1 with sample sizes by stream and year to the main text. We have done this now as Table 1, which should help clarify the differences in sampling across years.

Since you correctly point on that seeing genetic differentiation within CAC_S1, but not between CAC_S1 and CAC_S2, is confusing, we rephrased and added language that helps to clarify this point (lines 247-257).

We were also surprised initially by the small-scale genetic differentiation in *M. guttatus.* However, other studies *of* *M.* guttatus (not in hybrid zones) have found substantial population structure (and incredible amounts of genetic diversity) at small scales, so this is not unheard of -- we added a point about this, with references, to the discussion, lines 935-937. We don’t think severe inbreeding is responsible, since our selfing rates, which are broadly similar in the two streams (see Table 2), don’t suggest abnormally high inbreeding.

Your comment about Fig2A lower panel (now Fig2B), together with Reviewer 2’s comments on this figure, suggests that we were not sufficiently clear about the construction of this figure. We have now highlighted in the legend that this panel contains only *M.* guttatus and admixed individuals, with *M. nasutus* as well as all LM samples removed in order to focus on additional within-CAC variation. The additional PCA analysis requested by Reviewer 2 (see comments below), now provided as Figure S2, may also help to clarify this point.

*2. How many M. sookensis in this study and how many are allopolyploid? Line 183 says 11 from CAC_S2 with His ~0.5 were allopolyploid species M. sookensis and were removed from further analyses. I found 16 M. sookensis (9 from CAC) in Fig3 major haplotype. Table S1 has 11.*

Thanks for pointing out that we need to explain the source of the numbers in Figure 3 better. You are correct that we detected a total of 11 *M. sookensis* (all of which are allopolyploid). Only 9 of these were used in the chloroplast haplotype dataset (see the different filtering procedures in the *Organellar haplotype networks* section of the Materials and Methods, lines 1239-1242). These 9 were combined with an additional 7 previously sequenced *M. sookensis* for the chloroplast analysis. To avoid confusion, in the captions within Figure 3, we have now specified which samples are from our new dataset (bolding these) versus previously published data.

*3. Line 197 – 200 “In contrast, the haplotype is almost never found in M. guttatus, which has much higher organellar diversity … only two carry this CAC/LM haplotype”. I am not quite sure I understand this expression. I guess you mean the major haplotype is carried by all M. nasutus and M. sookensis but is rare in non-CAC M. guttatus with only two accessions (BOG10, DPR84). How about the 41 LM M.guttatus + admixed?*

Thanks, we see now that this wording was not clear. Our meaning here is that, outside of CAC+LM, the haplotype is rare in *M. guttatus,* appearing in only two accessions in the range-wide dataset (and one of these accessions is also from a contact zone with M. nasutus). Within CAC and LM, the haplotype is potentially fixed (all 41 samples of LM *M. guttatus + admixed* carry this same haplotype). We clarified this wording in the text (lines 291-299) and in the Figure 3 legend.

*4. Line 202 – 206 “We therefore infer that this haplotype is derived from M. nasutus.. is free from the effects of introgression”. I am not sure one can reach at these conclusions simply based on comparing the number of individuals carrying haplotypes in both species, but neither considering the effects of genetic drift nor selection on maintenance of introgressed alleles. For example, Ne could be much smaller in M. nasutus as it is selfing and thus introgressed allele can be fixed or lost at a much faster speed than the outcrosser.*

While it is formally possible that the haplotype is derived from *M. guttatus* and has introgressed into small-Ne *M. nasutus* before sweeping to fixation, we believe this scenario is unlikely for a number of reasons:

- The haplotype is present in multiple *allopatric M. nasutus* from different parts of the range, not just *sympatric* M. nasutus.
- The haplotype is absent from sampled *allopatric M. guttatus* (except possibly BOG10, sympatry status unknown).
- The haplotype is present in two very disjunct *sympatric M. guttatus* sites CAC+LM and DPR, ~1000km away, which would be a fantastic coincidence if not due to *M. nasutus -> M. guttatus* introgression.
- The direction of introgression from *M. nasutus* into *M. guttatus* is consistent with the primary direction of introgression in the nuclear genome and the asymmetrical nature of nuclear introgression.
- The direction of introgression is consistent with our finding (and previous work) that new *F1-like* hybridization, while rare, is slightly more common via maternal *M. nasutus* than via maternal *M. guttatus*.

*5. Line 213 “HI=0.1-0.2 for LM and 0.23-0.3 for CAC_S2” Is there any possibility of switching the values by mistake?*

Yes, this was a simple error swapping the values. It has been corrected, thank you for catching this! (Line 328-329)

*6. Line 228 “plot S1_5B, for example, … ” Is that simple because sample size is too small in year 2012 in this plot?*

This is a valid point, since the plot does have a small sample size. However, the phenological difference between 2012 and 2019 (shift to later flowering, see Figure S7B) seems to supports this being a real change, and is not subject to the same sample size issue.

*7 Line 417-421. The authors used all evidence to show the mating system and phenology difference caused asymmetric gene flow and I think that is a very neat explanation. On the other hand, no evidence in this study showed better adaptation of M. nasutus. One needs to explain more why cytonuclear incompatibility grants disadvantage to M. guttatus and admixed if the possibility of selection needs to be discussed. Comparing to selection, I think drift with small and large Ne in selfer and outcrosser could be more interesting.*

We agree that asymmetry due to mating system is the best hypothesis to explain the directional organellar capture. We decided to mention selection as a potential alternative hypothesis, but we’ve softened the wording to make it clear we are not trying to argue in favor of this option (Line 903). We don’t believe Ne plays as important of a role here, because the expectations would be opposite (small Ne in the selfer could lead to fixation of an introgressed haplotype in *M. nasutus*). And as discussed in the point above, we are confident through multiple lines of reasoning that the direction of the introgression is from *M. nasutus* into *M. guttatus*.

*8. It seems only precipitation data has been collected and compared. How about other environmental variables such as max or min temperature which is expected to affect phenology or the pollinators as well.*

This is an excellent point and suggestion! In response, we added publicly available temperature data to Fig 1 (now Fig 1C, and referenced in the Introduction at lines 154-156 and the Discussion at lines 972-973) for comparison: the 2022 flowering season appears to be a modest outlier in both precipitation and temperature. We do not include an exhaustive analysis of multiple environmental variables, because the number of years of sampling we have (at most three years of phenology data) is insufficient to run statistical tests with. As pointed out also by Reviewer 2, any conclusions about the role of specific environmental factors driving the fluctuations we see are speculative at this point, and would require additional years of data to test rigorously. We have adjusted our language in the Discussion (see lines 968-973, 992-996, and 1003) and Introduction (deleted sentence at line 171, changes to lines 175-177) to make it clear that these are hypotheses to be explored further and not conclusions of the current study.

*9 How many genetic loci were amplified? Are they random? What is the proportion of genome covered?*

Thanks for the reminder to add genome coverage info – in the original manuscript, we did not emphasize enough the low-coverage design of our sequencing methodology. In response to this and a similar suggestion by Reviewer 2, we have added summaries of genome coverage depth and breadth per sample to the Materials and Methods (lines 1118-1121). The numbers of genetic loci used in our genotyping and ancestry panels are also provided in the Materials and Methods (lines 1143, 1145, and 1151). We also added language describing the purpose and differences between the different loci panels (lines 1146-1149, 1153-1166). Finally, we added language to emphasize the low-coverage approach to both the Materials and Methods (line 1082) and upfront in the Introduction (line 157) and Results (line 183).

*10 The discovery panel is quite small, especially only four M. nasutus were sequenced, for allele frequency estimation. How does one guarantee there is no ancestral polymorphism exists even in allopatric sites? What is the average genetic distance (FST or Dxy) for two species? I think one at least should discuss the possibly consequences of mis-assignment of ancestry.*

We appreciate this point, which has an important bearing on the strength of our results and conclusions. We have added some additional information and justification throughout the Materials and Methods and Results to address these concerns, as follows:

*M. nasutus* (the selfer) has a much smaller genetic diversity than *M. guttatus:* synonymous pi is ~1.1% for *M. nasutus* across distant populations, while range-wide synonymous pi for *M. guttatus* is ~4.9%. On the other hand, divergence between the species is on par with within-*M. guttatus* range-wide diversity (synonymous dxy is ~4.9%). These values are previously published in Brandvain et al. 2014 (<https://doi.org/10.1371/journal.pgen.1004410>). As a result, we need a smaller number of *M. nasutus* reference samples to effectively identify *M. nasutus* alleles, while a larger number of *M. guttatus* samples is necessary to have confidence that alleles are present in *M. guttatus*. (Added a note with this info and reference to Materials and Methods, lines 1132-1134).

Indeed, many alleles that are ancestry-informative (high allele frequency differences between species) are not 100% fixed in *M. guttatus*, and ancestral polymorphism is definitely present. For our ancestry-informative sites, we therefore used an 80% frequency difference cutoff (Materials and Methods, line 1151) to define ancestry-informative sites. The Ancestry_HMM approach to assigning ancestry is designed to account for this uncertainty, because it integrates across multiple sites when assigning ancestry probabilities (reasoning added to Materials and Methods, lines 1154-1166).

Still, there is always a possibility of mis-assigned ancestry. Since we primarily use ancestry averaged across the whole genome in this study, we are less worried about occasional errors at any particular site, which should average out. A bigger worry would be if there is a systematic bias in errors favoring one or the other parent. We have a few reasons to think this is not a major concern in our data, which we have also highlighted in the text as follows:

- The identification of allopolyploids, while not planned, provides a useful check as they have an expected ancestry contribution of 50/50. We see that our *M. sookensis* allopolyploids have consistent ancestry values ~0.47, showing only a very slight bias. (See lines 281-283)
- Average ancestry values within a plot are stable across years, providing confidence that ancestry inference is reliable on repeated samples. (See lines 331-337 and 361-362)
- We inferred ancestry in a previously characterized set of 8 high-coverage admixed samples from CAC and found similar levels of ancestry to previous results. (See lines 1200-1206).

**Reviewer #2: Review of “Fluctuating reproductive isolation and stable ancestry structure in a fine-scaled mosaic of hybridizing Mimulus monkeyflowers”**

*Summary of research:*

*This study investigates hybrid population structure and reproductive isolation between M. guttatus and M. nasutus. The authors combine genetic data from wild individuals and greenhouse-reared offspring with phenological data to explore how phenology and mating systems influence introgression dynamics across years. The study contributes to the growing literature documenting various outcomes of hybridization. They empirically detail how variation in phenological isolation of M. guttatus and M. nasutus across years corresponds with admixture frequency. The study also quantifies how differences in mating system (selfing vs. outcrossing) result in strongly directional gene flow - with the selfer admixing much less frequently, as predicted by theory. They find that ancestry proportions generally remained stable in studied populations.*

*While none of the claims are particularly novel, the work provides uncommon empirical validation for commonly discussed theory (eg that phenological isolation impacts hybridization rates). The sequencing of maternal-offspring pairs is a particularly nice component of the study.*

*Major comments:*

*In general, the naming and use of different sites is difficult to follow (For example, why are there only a few plots with A and B?). Consider ways to simplify/clarify more often why you are focusing on specific sites. Further, please address what biases the imperfect sampling scheme may cause in your analysis and how you address it. If space allows, also consider moving table S1 of sampling info into the main text.*

*In general, given the difference in timing of sampling, sampling effort, climate, and sequencing batches for each plot, more detail is needed in the reporting of the results to clarify how sampling ‘design’ influences methodology and interpretation.*

We thank you for the careful comments and useful suggestions! We discuss each point in detail below.

We appreciate your point that the plot names were confusing. The use of plots with ‘A’ and ‘B’ in the names was for internal reasons related to future sampling. To alleviate any confusion, we have opted to simplify the plot naming. Now, all plots are named sequentially from South to North within a stream, e.g. 1 to 8 for CAC_S1. We chose South to North for consistency with previous published work at CAC (Kenney and Sweigart 2016).

Your comment about the sampling design being difficult to follow are strongly headed. We made multiple changes to highlight the specifics of site vs. year sampling and increase clarity:

- In the Introduction, we now lay out explicitly the difference between our broad spatial sampling (three streams) and our deeper temporal sampling (which does not cover all three streams) (lines 157-177).
- At the start of the Results, we provide sampling numbers and lay out which streams were sampled in which years (lines 183-187).
- Following your suggestion, we moved Table S1 with sampling details into the main text of Results.

In addition, following your suggestions about further exploring variation between the streams, we made the following changes to our analyses and results:

- We added a plot-level breakdown of hybrid indexes for CAC_S2 and LM to Figure 4 (now 4B-C), where this had previously been a supplemental figure. The previous panels in Figure 4, with plot-level phenology, have been moved to Figure S7 instead. Figure S8 has been added with comparable phenology information for CAC_S2, parallel to the information in Figure S7.
- For selfing rates, phenological comparisons, and calculations of reproductive isolation, we now always separate samples/analyses by stream and discuss CAC_S1 and CAC_S2 separately. See especially Tables 2 and 3; examples of changes in the text include lines 470-480, 632-639, 913-935, and others.
- Stream (CAC_S1 or CAC_S2) is now tested as a potential explanatory variable in all of our statistical models (See Table S2).

*Include a supplementary table of sequencing batch information and clarify in the text how you can be sure batch effects are not driving any of your results (e.g., clarify that PCA clusters on PCs 2-4 do not align with sequencing batch (?) to ensure they are biologically relevant clusters).*

Thank you for pointing this out, we were not sufficiently clear about the sequencing batch information in our original manuscript. We agree that it is important to check for the effects of batch on our analysis. We added a description of the different batches we sequenced to the Materials and Methods (lines 1094-1103). Batch 1 was a small test batch including 2019 maternal samples only. Batch 2 included all remaining 2019 maternal and offspring samples plus all 2021 samples. Batch 3 included all 2022 samples.

As it turns out, because of our sequencing approach, batch is associated with sampling year. Since we include year in our figures and models, it did not make sense to include batch as a separate effect. However, as you point out, it is still important to check that year (and therefore batch) does not drive our population structure or ancestry results. We highlight points in the text where we confirm that this is not the case, detailed below:

- Both within plots and within streams, the distribution of ancestry is consistent across years – this implies that there are not strong batch effects in our ancestry calls. This is now addressed explicitly in lines 334-337.
- All of our PCA clusters include samples from multiple years (except the LM cluster because LM was only sampled in 2021). Year does not drive any of the signals in our PCA, and therefore neither does sequencing batch. This is now highlighted in the Results, lines 254-257.
- In the PCA, despite small sample sizes for 2021 at CAC, 2021 CAC samples do not cluster with 2021 LM samples and instead group with CAC samples from other years. This shows that the LM cluster is not a year/batch effect.
- We do show that there are differences across years in offspring HI shifts (i.e., Figure 5). But these involve comparisons between parents and offspring, which were almost always (except a small number of 2019 parents) sequenced in the same batch, so batch effects should not drive these differences.

*As written, there are several places where results give the impression of avoiding discussion of data that does not support the central arguments. I have left line-specific comments below about where this is most notable in the results. Beyond a lack of clarity about missing data from certain sites or years mentioned above, the discussion of CAC_S2 data (and LM data) is minimal. This feels misleading and seems like a missed opportunity to clarify what about these two streams causes different admixture and population structure outcomes.*

We appreciate this comment -- this impression is a major concern, and we do not want to imply that we have selectively avoided discussing some results. We have made changes to the analysis to more explicitly explore differences between the streams (see response to the earlier comment above). In addition, we have made a number of changes to the language throughout the manuscript that should alleviate these concerns. The most important of these are detailed here; we also direct you the individual responses to your line-specific comments further down.

- Some of this concern can be addressed by more clearly laying out the sampling scheme and the differences in sampling across the three streams. As noted in the comments above, we moved the supplemental table with sampling details to the main text (now Table 1).
- We also revised the end of the introduction to be clear about the differences between between our breadth (three streams) sampling and our deeper temporal sampling (which does not cover all streams equally) (lines 157-177). In addition, we place this sampling information in the beginning of the Results (lines 183-187).
- Table 2 is now split into CAC_S1 and CAC_S2 data, treated and discussed separately (see lines 438-439).
- Table 3 is now split into CAC_S2 and CAC_S2 calculations. The gaps in information within Table 3 highlight where the limitations of our sampling prevent us from making stronger claims about reproductive isolation in CAC_S2.
- We describe in the Results (lines 472-478) why it is difficult to make certain calculations of reproductive isolation between admixed and *M. guttatus* cohorts within CAC_S2: there is a lack of population structure by either hybrid index or plot, meaning that any criterion for grouping individuals to calculate reproductive isolation between them is arbitrary, and our limited sampling of *M. nasutus* families further reduces the number of comparisons we can make. However, given your suggestions, we still provide the calculations where possible, along with this caveat. These and other limitations are reiterated in Materials and Methods, lines 1302-1305 and 1326-1329.
- LM had similar limitations to CAC_S2 regarding measurements of reproductive isolation. Not only is there a lack of three-cohort structure, but *M. nasutus* is not spatially segregated from admixed individuals, leaving no clear hybrid index or plot-based structure (See Figure 4C). For LM, we also do not have any phenological data at all (none was collected in 2021, the only year in which we sampled LM) – this is noted at line 1057. Therefore, calculating phenological isolation is not possible for LM. We did not calculate selfing rates because almost all maternal families we sampled would be from the same ‘admixed’ cohort (and this cohort delimitation would be arbitrary anyway), so there were no meaningful comparisons to make across cohorts. These limitations are described now in the Materials and Methods, lines 1305-1309. We therefore focus on CAC only for all results and discussion regarding reproductive isolation.
- As noted in previous comments above, we clarified which results and conclusions are derived from which streams throughout the manuscript.
- For the following figures which have data presented from both CAC_S1 and CAC_S2, we separated the model fit lines and confidence intervals to differentiate the two streams: Figure 4E (individual hybrid index by flowering date), Figure 5A-B (offspring HI deviation by maternal hybrid index), and Figure S5 (selfing rate by hybrid index). For all statistical models, we tested the effect of stream on the model fit (detailed in Table S2).
- We revised the discussion to highlight where certain conclusions pertain to only certain streams (e.g., lines 914, 963, and elsewhere), and to make comparisons across the streams where possible (e.g., lines 916-935).

*The authors claim to address how the genetic structure of admixed populations is maintained over time, but their data do not directly address this question. They clearly outline a hypothesis in the discussion as a future direction - investigate microsite differences that may be responsible for differences in phenology and, thus, flowering time overlap and admixture. However, this paper does not address this directly, and any such claims should be removed. The authors should instead focus on more thoroughly exploring the results of this study and the differences in data across sites that are poorly addressed. For example, I don't believe the data from LM are even mentioned in the discussion.*

These are important points, and we do not mean to over-sell our results. We adjusted the language in the Introduction and Discussion sections to more clearly differentiate between direct conclusions of this paper (for example, the existence of ancestry structure and variation in reproductive isolation) and more speculative hypotheses about the causes and effects of these patterns (especially regarding the influence of environmental factors on fluctuations, the presence of microhabitat differences, and the effect of fluctuation on stability over time). See examples of changes at lines 870-877, 933-935, 943-946, 992-996, 1003. We believe these hypotheses are worth discussing and are important to the broader relevance of the study, but we agree they are not direct conclusions of the study and are instead avenues for further exploration.

We wish we had more conclusions to draw about LM – but see our previous comment above about the limitations of our LM data. We do highlight a few places where LM data are used to draw conclusions (e.g., lines 849-851, 889-890, 916-917), and we make some explicit comparisons among the three streams, including LM, in our revised Discussion (especially lines 916-935).

*Minor comments:*

*In the paragraph starting on line 125, you do a good job explaining how M. nasutus habitat and enviromental fluctiations impact flowering time and life history, but lack a comparison to M. guttatus. I think it is important to clarify here (with citations) how this impacts phenological overlap with M. guttatus as this is critical background for the reader to understand your conclusions.*

We added a sentence here (line149-152) focusing specifically on the phenological overlap aspect, and generally rewordd this section (lines 144-156) for clarity.

*Lines 139-141: This sentence is unclear. For example, it is not clear what you mean by “three additional years”, and I am left wondering whether sampling was different at the two sites. Why is one site named and the other not? Why did you do additional sampling at a ‘nearby site’? How do you know they were offspring - were they tracked in the wild, or grown in a greenhouse? If greenhouse grown, are you breeding them in the greenhouse, or collecting new seed from the field each year? While these details are in the materials/methods, given the results first format of this journal, you need to better clarify the experimental/sampling design up front for readers who may not read your methods first to be able to understand your results. Further, this description is hard to reconcile with the caption of Figure 1, where you define 3 sites. Please make sure you are consistent with how you describe the number of sites and sampling effort.*

We have significantly revised this section (now lines 157-177) to increase clarity on our sampling scheme and how it is utilized – in particular, highlighting that we don’t have multi-year data across every stream, but that the multi-year data is restricted to CAC_S1 (and partially CAC_S2) but complements the spatial breadth of data from all three streams. We also clarified the use of offspring here (lines 168-169).

*Methods:*

*520-521: It is not clear what is meant by the parenthetical here, especially the “1-3 individual fruits per maternal family” piece, even after looking at supplementary table S1. Please make the sampling/sequencing design more clear/explicit.*

We restructured previous table S1 and moved it to the main text (now Table 1) to help clarify this. We also adjusted the wording of the text (lines 1062-1066) to increase clarity, and referenced Table S3 which has additional details on offspring sampling.

*542 states that samples were sequenced across 3 runs but does not reference a table showing which samples were in the same run. This can be important for batch effects if one run contains all of one species or sampling location, etc. Please include information on what samples were grouped in the same sequencing batch and whether you see clustering in PCAs by batch (eg do samples of the same species ever cluster by batch), and how batch effects might influence your data interpretation.*

We added information at lines (1094-1103) detailing the sequencing batch information. See our response to your comment above where we address potential batch effects.

*544-549: please report standard genome coverage metrics (breadth and depth of coverage) rather than raw read numbers, which are not meaningful without reference to genome size, etc.*

Thanks for the reminder to include actual genome coverage metrics. We have now included these in the section on reference mapping (lines 1118-1121). We also added some wording to highlight the low-coverage nature of our sequencing, which was a design choice that we want to be clear about (lines 1082, 157, and 183).

*Starting on line 565, translate filters into plain English. Readers should go to the co-published code for the specific parameters (and that GitHub repository should be cited in the methods).*

We have referenced the GitHub repository here now, and simplified the discussion of filters here (lines 1138-1142) and elsewhere (e.g., lines 1189-1192) in the Materials and Methods.

*571-576: Please further clarify the differences between these two panels and why they were created. Are many of the sites in the second ancestry informative panel not covered in at least 60% of your test samples?*

Thanks for these comments – we realize we were not clear about the different purposes of these panels and how they relate to the low-coverage nature of our data. We added text clarifying the purpose of each of these panels and how they differ (lines 1146-1149 and lines 1153-1166). Yes, many (most) of the ancestry-informative panel sites have less than 60% coverage in the test samples; this is a consequence of the low-coverage approach we took to sequencing (see our average genome coverage of 0.48 per sample per site, line 1121). We note this here in the text, along with a justification for the utility of combining information across many lower-information sites to estimate ancestry (lines 1153-1166).

*The inclusion of a test for the ancestry calling (lines 608-614) is a well-executed and important check! Nice work.*

Thanks!

*665-668: please state how many were excluded (not just how many were kept) to give a clear picture of how frequent this was. It would also be nice to know why the authors think this happened as if it is a substantial number, it might indicate that mix-ups happened in sampling/sequencing that could mean mismatched families and offspring with matching parent types were kept (ie looked fine in terms of coming from the right parents, but might be mislabeled in terms of family). The authors should clarify this point carefully to assure readers that the retained data are trustworthy.*

You raise an important point about potential mix-ups, and we realize we did not lay out the rationale for this filter before. The relevant numbers have been added (initial numbers in lines 1258-1259, and final numbers with proportions in lines 1271-1272). We retained 79% of initial offspring and 60% of maternal families after this filter.

In lines 1272-1279, we lay out the rationale behind this relatively strict filter, and why it is set up to remove samples or families with poor ancestry inference, rather than necessarily indicating mix-ups in sampling. We elaborate on our rationale for choosing a smaller number of high-confidence families here:

- We were mostly concerned with aggregate selfing rates, which could be biased by poor quality, rather than the specifics of individual families. A smaller number was still sufficient to obtain these aggregate values.
- Due to the low-coverage nature of our data, correctly labelled samples might have errors at specific loci. For calculating hybrid index, these errors should average out – but the estimation of selfing is quite sensitive to patterns at a small number of loci. Therefore, removing samples with high error rates should limit our misspecification of selfing rates.
- The selfing rate estimation program (BORICE) rapidly became unmanageable with larger numbers of offspring samples (run times lasting weeks without finishing), so a smaller number of high-confidence samples was more feasible.

*681-681: Explain why this was only done for CAC_S1 (lack of 3 distinct cohorts, yes, but there is admixture that could be explored in CAC_S2, eg you could still quantify RI between the two species)*

Based on your comments, we decided it was best to include all possible RI calculations for both CAC_S1 and CAC_S2, even though the data are less complete for CAC_S2. We now include a calculation of mating system RI and phenological for CAC_S2 for the years and comparisons where this is possible (Table 3). We point out in the Results (lines 472-478 for mating system and 744-746 for phenology) some limitations of these CAC_S2 calculations.

*694-696: why was only one representative plot from one site chosen to represent the three groups? It seems like the phenological value should be calculated from data from all individuals in each cohort as was done for mating system. It currently gives the impression that those plots could have been cherry-picked for clear differences in flowering time. Either give a clear justification for only calculating phenological RI across those 3 plots (maybe because of the best consistent sampling as implied in the results?), or do the analysis across the full set of individuals per cohort (or at least all individuals per cohort in CA_S1 where flowering time is consistent within cohort across plots).*

This is a good point! Following your suggestion, we now calculate phenological RI using as all plots that have a single primary ancestry cohort (six plots for CAC_S1, and seven for CAC_S2). We still exclude two plots from CAC_S1 because they contain multiple ancestry cohorts, so the plot-level phenology data cannot be assigned to a single cohort. And we do not calculate CAC_S2 phenology for 2012 because we don’t have any plots with only *M. nasutus* in that year. We describe these choice and the plot assignment in Materials and Methods (lines 1320-1328).

*Line 712: This is a great aspect of your study; using offspring to look at assortative mating is cool and interesting!*

Thanks!

*724: If you see batch effects in a PCA (clustering of samples by library or sequencing run), you should include batch as a random variable. (true for all regressions in your analyses)*

See earlier comments above where we address the potential for batch effects.

*727: “for only families with maternal HI<0.5.” Remind readers what this means (what does HI<0.5 indicate)*

We added a note on the reasoning for this in the text (line 1386).

*Results:*

*170-171: Please add information about how this relates to physical distance as you did with the description of physical distance and genetic distance for CAC-1. Also, it seems possible that some of this structure could be related to batch effects given the relatively low variance explained by PC axes 2-4 and the lack of differnetiation between the 2 CAC streams when there is differentiation btw plots from a single stream. Again, please address this by clarifying which samples were in the same library preps/sequencing runs so it is clear that PCA clusters are not simply aligning with sequencing batches.*

We re-worded this section (lines 247-257) and added the physical distance information, highlighting the contrast between within-stream differentiation and a lack of between-stream differentiation.

See response to earlier comments above about potential batch effects.

*200: Why I this surprising? If you are going to see the nasutus/sookensis haplotype anywhere in guttatus, wouldn’t you expect it to be in a sympatric site?*

Agreed. We removed the word ‘surprising’ here (line 299).

*212-215: You report the same HI peak ancestry for the CAC-S2 guttatus cohort and the CAC_S1 admixed cohort; this is also clear in figure 2C. How can the same hybrid ancestry be called the ‘admixed’ cohort in one location and guttatus in the other? I understand calling 3 different cohorts within CAC_S1, but ncestry cohorts should be defined by consistent ancestry proportions, and therefore the CAC-S2 peak at HI .25-.3 should be called part of an admixed cohort.*

Thanks for catching this: as also pointed out by Reviewer 1, these numbers were erroneously swapped. We fixed this mistake, and also clarified the language, referring to the CAC-S2 group as admixed (lines 328-329).

*215-216/219-220: Please clarify that this is across years for the 2 streams for which you had multiple years of data, and that you cannot confirm the patterns have been stable across years at the LM site. The current writing implies that your results show consistency across all three streams through time.*

We adjusted the wording to focus on CAC_S1 for all discussions of stability over time: here (lines 331-337), elsewhere in the Results (lines 361-362), and in the Discussion (lines 913-916).

*217-218: Here you contradict the previous statement and properly label the second CAS-S2 cohort as admixed. Please just adjust lines 212-215 to match this classification.*

Done, see above comment.

*221-230: This is cool and interesting! However, please clarify why you are only looking at CAC_S1 (presumably because there is no spatial structure in S2). I think the study would be strengthened overall by a more thorough treatment of both streams and how they differ. Focusing on how and why you think these streams differ would be a more honest interpretation of your data than building a story about stable ancestry mosaics through time due to microhabitat variance when you do not have data on microhabitat. You do have lots of great genetic data, from multiple sites! Focus on this and fully interpreting it.*

We appreciate this comment and the interest in comparing across streams. As detailed in the responses above to earlier comments, we updated our analyses to separate out the effects in different streams (for example, Tables 2 and 3) and all statistical models in Table S2).

*233: Again, interesting approach and results. However, here you go back to using data from both streams, even though the question being tested is based entirely on results from one stream. The reasons behind these choices of when to include different sites in the analysis need to be better clarified throughout.*

As detailed above, we now explicitly separate out the two streams for this and other analyses.

*242: Rather than ‘a small number’ explicitly state how many*

Thanks, we include this number now (it was six) in line 435.

*306-307: This really is an awesome component of your study!*

Thanks!

*Discussion:*

*359-361: This logic feels at odds with your data/results; your plots vary in ancestry (mosaic of ancestry), but your results do not actually investigate the mechanisms underlying this differentiation. You do not look at microhabitat differences (moisture etc), and do not calculate RI across all plots, so you can’t make a statement about fluctuating RI and its relationship to admixture across the landscape. Further, the plots at the second stream, CAC_S2, presumably do not vary in ancestry across the same spatial scale, though this result was largely ignored. Your more nuanced explanation on 390-394 is great and clarifies that this (very reasonable) speculation about causation is grounded in the existing literature, not your results. Please revise this early statement and more clearly connect it with your actual data/results.*

We agree that this sentence is more related to future directions and hypotheses than the existing data. We removed the offending sentence (line 833) and reworded the following sentence (line 834) to emphasize what we do know.

*427-429: This statement would be bolstered by a more careful explanation of results from CAC_S2. If distances are the same but there is no population structure in S2 compared to S1, this also indicates that factors other than distance are responsible for the (admixture based) population structure in CAC_S1.*

This is a good suggestion! We clarified that this statement refers to CAC_S1 (line 914) and added some discussion contrasting CAC_S1 structure with a lack of structure in CAC_S2/LM, later in the paragraph (lines 916-935).

*433-435: This feels misleading given that you did not explore the differences in microsite conditions and how they relate to differences in reproductive isolation, which is what you are describing as the factors likely responsible for a consistent mosaic of admixture.*

We rephrased this sentence to focus on the differences in reproductive isolation that we are directly measuring, rather than discussing microsite conditions that we do not address (lines 943-946) We note that the barriers we measure are likely not the only important forces, and that microsite differences probably play a role too (this is an avenue for future study).

*Figures:*

*Figure 1B - At a minimum, please explain in the legend that colored lines are collection years, and grey is other years for context about precip variability. However, I recommend coloring and labeling all years, as in some cases, the environmental conditions of the preceding year are very important (reflecting selection on parents and produced seeds).*

We appreciate the point that previous years may be important for selection effects. We did adjust this Figure (now Figures 1B-C), in response to Reviewer 1’s suggestion above, to include both temperature and precipitation, and to show at finer resolution the heterogeneity in timing across the season. However, we decided to keep the other years in grey because including 13 separate colors was extremely cluttered and difficult to read. We did clarify the use of colors in the figure legend. Since we are presenting this figure as an example of heterogeneity and not trying to draw a causative conclusion about the role of a specific factor, we believe this is sufficient. A more explicit test of the role of precipitation on selection would need to dive deeper into the effects of preceding years.

*Figure 2 - Please clarify that LM and NAS were only removed visually, but were still part of the PC analysis that generated the PC3 and PC4 values.*

*In fact, if the point of showing PCs 3 and 4 is to examine patterns in just CAC, and visually exclude LM and NAS, you should do a separate PCA of CAC so that variance explained by the PCs is meaningful for understanding within CAC divergence.*

*You can include PC3 and PC4 for context in the supplement about how much pop variance there is in CAC relative to divergence with NAS, but since that is not a primary/important component of your results, I would recommend 2 separate PCAs instead. At the least, the PCA without NAS and LM should be run to ensure structure within CAC is consistent with the structure indicated by PC3 and PC4 in the larger analysis. It is also unclear in this why you specify in the legend (and not the figure itself) that LM-A was removed (what is LM-A and how is it subsetted from LM?)*

We appreciate this suggestion and opportunity to view our data in new ways. In response to this suggestion, we ran a separate PCA of CAC only (with LM and *M. nasutus* removed). This new analysis largely agrees with the results of the original analysis, and is now presented as Figure S2.

We chose to present this new analysis as a supplement and retain the original analysis as the main figure, for simplicity and because the original analysis highlights a few important points: the relative importance of ancestry (PC1) compared to non-ancestry structure (PCs 2-4), and the consistency of the structure across years (which validates a lack of batch effects).

We clarified in the legend and directly in the figure that LM and NAS were only removed visually for PC3 and PC4, as well as making this point in the Results text (line 225).

*Also, if you are going to include the allopolyploid, you need to explain why - what does inclusion add? What are the progenitor species, etc. (state in legend that it is allop btw nasutus and guttatus)*

Good point: we now note in the legend that the allopolyploid has an expected 50/50 genome contribution from M. guttatus and M. nasutus, which provides a useful check for our ancestry assignments.

*2B Is a nice visualization of the directional admixture!*

Thanks!

*2C - It looks like lower admixture in CAC-S2 could be largely due to demography and there being fewer overall nasutus. Please address this - is there less nasutus at S2?*

Demography could definitely play a role in lower CAC-S2 admixture. However, our samples do not necessarily reflect the relative abundance of nasutus vs. guttatus, since this depends on the placement of plots, plot density, and the number of samples we took from each plot. In fact, we likely under-sampled nasutus because we were focused on capturing the variation among admixed individuals, and we expected nasutus to have little variation. In 2021, we under-sampled CAC nasutus even more because we arrived to the site late, after most nasutus had flowered. We added a sentence to the discussion that addresses this point (lines 872-875).

*It is confusing trying to follow which species/sites were sampled in which years. Please rewrite the legend and/or clarify with a visual like a small table showing site and the years with data for that site.*

Thanks for pointing this out. We added at the start of the legend a clarification of sample sizes and which sites were sampled in which years. The addition of Table 1 in the main text should help with this as well.

*Figure 3: not everyone will be familiar with a NJ-Net Haplotype Network; please describe in the legend what each circle and its size represent, as well as the meaning of the labels and the hash marks on joining lines. This may sound silly/seem obvious, but it will help unfamiliar readers orient more easily to the figure and the key finding it shows.*

This is helpful advice! An expanded description of the network has been added to the legend.

*Figure 4: It would be more intuitive for the labels to go from lowest number at the top rather than largest (S1-1 to S1-6 rather than visa versa). Why is S1-1 divided into A and B, but not the others?*

The ‘A’ and ‘B’ notations were present for internal reasons (involving consistency with future sampling plans), but we realize that this was unnecessarily confusing. We have updated the plot names to a simple numbering scheme (1 to 8 for CAC_S1, 1 to 7 for CAC_S2) to avoid confusion. We have, however, elected to keep the bottom-to-top numbering because it is consistent with previous published data from this site (Kenney and Sweigart 2016).

*The legend makes it sound like some plots are purely guttatus, some are admixed, and some are purely nasutus. Consider rephrasing for clarity, somethign like, “plots X, Y, and Z with primarily nasutus indivisuals have earlier flowering times”.*

The wording has been adjusted according to this suggestion.

*4 - The legend states that pops with admixed individuals have later flowering (intermediate) than M. nasutus, and that M. guttatus has late flowering. While this appears true in 2022, it does not appear completely accurate across all years (eg. 2019, S1-1 is later than S1-2, S1-5B was late in 2012, etc). Your other plots and analyses show this relationship more clearly, let those speak for themselves.*

In response to your other comments and suggestions, we have re-organized Figure 4 to highlight and compare all three streams. Previous panels 4B and 4C are still provided but are now a supplementary figure (Figure S7, with an associated CAC_S2 figure added as Figure S8). This change also allows the phenological discussion to focus on figures 4D and 4E, as you suggest.

*4D - Dotted lines are nearly impossible to see; please specify quantiles for the interquartile range (is it the standard 25th-75th percentile?).*

We previously had both interquartile range and full (0-100%) range depicted, but the multiple types of lines were difficult to see, as you point out. We updated this figure to focus on the medians and the range of flowering, because this better highlights the change across years in phenological overlap. The new Figure 4D is simpler and focuses on the most relevant information.

*4D/E - These are much more convincing plots for making the point alluded to in the description of 4B! Remove commentary from 4B in favor of these analyses.*

Previous 4B is now Figure S7 – we have removed the associated commentary from that figure legend and simply described the figure, allowing 4D/E to show this relationship.

*4E - It looks like the relationship is different for CAC-1 and 2 in 2022, where the relationship seems weaker for CAC-2. It is misleading to lump CAC-1 and 2 for 2022 when you also plot 2019, but only CAC-1. Please either include separate trend lines for CAC 1 and 2. This plot and the claim of association between hybrid index and open flower date needs statistical support - please include a reference to the stats in the legend. Also, include a confidence interval around trend lines to visually represent the variance in the trend.*

Thank you for pointing this out -- you noticed an important difference between the streams that we had not highlighted. We now provide separate trend lines (and confidence intervals) for CAC_S1 and CAC_S2. This shows that the relationship is similarly strong in both 2019 and 2022 for CAC_S1, but much weaker in CAC_S2. This change is also reflected in the associated statistical models (Table S2) and how we present this figure in the Results (lines 632-639).

*Figure 5 - The labeling within the plot is very helpful here! The first part of the second sentence in the legend is confusing. B is cool and interesting, but the blue and orange confidence interval shading are difficult to see. Please darken the shading in those colors or add a boundary to the shaded area.*

Thanks for the suggestions! We updated this figure to separate out the effects of stream (CAC_S1 or CAC_S2). We also darkened the confidence intervals, and stretched the figures lengthwise to make these details easier to see. We adjusted the wording of the second sentence for clarity.
